# Portrait of a Zoo Bristol Zoological Gardens 1893–1985

**Published:** 1985-07

**Authors:** 


**PORTRAIT OF A ZOO**
**BRISTOL ZOOLOGICAL GARDENS**
**1893–1985**
**Robert and Anne Warin**
**assisted by the Director, Geoffrey Greed**
**Redcliffe Press, Bristol. Price £2.95**

Robert and Anne Warin have produced a delightful book to celebrate the 150th Anniversary of the Zoo. Since its early days, Bristol Zoo has excited the enthusiasm and enlisted the help of Bristol's doctors and Dr. Warin is one of a long line. He has been an active member of the Council for many years and many of us will remember that 'Medicine and the Bristol Zoo' was the title of his Presidential Address to the Bristol Medico-Chirurgical Society in 1981. Everyone who loves Bristol Zoo – and that must be everyone who has ever visited it – will find the book compulsive reading. Though it is a popular book and intended for everyone it is nevertheless full of Zoology as well of good stories, it is also a serious account of the problems the Zoo faces in keeping its finances as well as its animals healthy. That the director of the Zoo, Mr. Geoffrey Greed has given much assistance is clear from the wealth of anecdote that the book contains. It is beautifully illustrated and produced and at £2.95 is a real bargain. It is also the perfect souvenir to give one's children and grandchildren when one has taken them around the Zoo – which is perhaps when most of us most often go. Buy the book, read it and then go round again – you will see more than you have ever seen before – well, anyway I did.

